# PACS-Integrated Artificial Intelligence-Based Software Tool for Rapid and Fully Automatic Bone Fracture Assessment of the Tibial Plateau

**DOI:** 10.3390/diagnostics16203325

**Published:** 2026-10-13

**Authors:** Niklas Tuttle, Denis Zainullin, Mark Heyland, Julian N. Zierke, Alaa Bejaoui, Heiko Tzschätzsch, Felix Balzer, Hongyou Zhou, Marc Toussaint, Khaled Bousabarah, Sebastian Hölzl, Georg N. Duda, David Alexander Back, Timo Alexander Auer

**Affiliations:** 1 Center for Musculoskeletal Surgery, Charité–Universitätsmedizin Berlin, Corporate Member of Freie Universität Berlin and Humboldt-Universität zu Berlin, 10117 Berlin, Germanydavid.back@charite.de (D.A.B.); 2 Department of Radiology, Charité–Universitätsmedizin Berlin, Corporate Member of Freie Universität Berlin and Humboldt-Universität zu Berlin, 10117 Berlin, Germany; denis.zainullin@charite.de (D.Z.);; 3 Julius Wolff Institute, Berlin Institute of Health at Charité–Universitätsmedizin Berlin, 10117 Berlin, Germany; 4 Institute of Medical Informatics, Charité–Universitätsmedizin Berlin, Corporate Member of Freie Universität Berlin and Humboldt-Universität zu Berlin, 10117 Berlin, Germanyfelix.balzer@charite.de (F.B.); 5 Learning and Intelligent Systems, Technische Universität Berlin, 10587 Berlin, Germanytoussaint@tu-berlin.de (M.T.); 6 Visage Imaging GmbH, 12163 Berlin, Germany; 7 Department of Diagnostic and Interventional Radiology, University of Tübingen, 72076 Tübingen, Germany

**Keywords:** artificial intelligence, fracture detection, tibial plateau fracture, image segmentation, Picture Archiving and Communication System (PACS)

## Abstract

**Background/Objectives:** Tibial plateau fractures (TPF) are complex intra-articular injuries requiring accurate reduction and stable fixation; inadequate planning can prolong recovery and increase osteoarthritis risk. Although CT-based 3D reconstructions are routine, automated 3D identification and separation of individual fracture fragments is uncommon. We evaluated a PACS-integrated AI workflow for automated 3D segmentation of intact bones, fracture detection, and fracture-fragment delineation. **Methods:** This single-center retrospective study analyzed preoperative TPF CT scans acquired on 64- and 128-slice scanners. Femur, patella, tibia, fibula, and fracture components were manually segmented in Visage 7 by a radiologist (9 years of experience) and an orthopedic surgeon (4 years). Segmentations were exported as NIfTI and used to train a 3D nnU-Net model with default preprocessing and parameters on 20 annotated TPF cases using a two-phase training strategy (initial training followed by retraining with refined labels). The trained model was integrated into Visage 7 and executed via an interactive PACS button for fully automated segmentation. Two independent readers rated outputs on a five-point Likert scale for intact bone segmentation, fracture detection, and fragment delineation; inter-reader association was assessed using Spearman and Kendall correlations. **Results:** In 108 fully rated cases, intact bone segmentation scored very high (means 4.67/4.72; median 5) and fracture detection was good (means 4.15/4.20; median 4). In contrast, individual fracture-fragment differentiation was limited (means 2.64/2.75; median 2). Inter-reader association was very high (Spearman r 0.943–0.967; Kendall’s tau-b 0.875–0.971; all *p* < 0.001). Greater fracture complexity was associated with poorer fracture-fragment differentiation. **Discussion:** A PACS-integrated AI workflow can reliably segment intact bones and detect TPF within routine imaging, supporting rapid 3D visualization for surgical planning. However, robust separation of individual fracture fragments remains the primary limitation, motivating larger and more diverse training datasets and continued model refinement.

## 1. Introduction

Complex intra-articular fractures present a significant challenge for orthopedic surgeons. Among these, tibial plateau fractures (TPF) are particularly difficult to manage due to their complex morphology and involvement of the knee joint. Treatment is usually via open reduction to regain joint congruity and internal fixation, as articular step-offs have been shown to increase intra-articular pressure [1]. Nevertheless, articular malreduction is not uncommon, occurring in up to one-third of cases [2]. In cases of suboptimal reduction patients face prolonged recovery times and a significantly increased risk of early-onset osteoarthritis [3,4]. Therefore, accurate identification of individual fracture fragments and their positions is critical for surgical planning and for successful joint line reconstruction [5].

To assist surgeons in visualizing fracture situations and improve decision-making, CT reconstructions have become standard for complex fractures as they appear to aid in achieving anatomic reduction [6,7,8]. Modern TPF classification systems are increasingly based on CT scans (as opposed to two-dimensional X-rays) to guide decision-making regarding surgical approach and implant placement [9,10]. Three-dimensional visualization strategies have been increasingly utilized through virtual models or 3D-printing to facilitate the understanding of complex fracture patterns [11]. Artificial intelligence (AI) has shown promising results when applied to the detection of fractures in conventional X-rays as well as CT scans [12,13,14]. Moreover, semi-automated approaches have been described for fracture-fragment mapping [15]. However, there is currently no fully automated CT-based solution to recognize the individual fracture fragments in three dimensions and distinguish them as separate volumetric elements.

The aim of this study was to evaluate a Picture Archiving and Communication System (PACS)-integrated AI-based workflow for the automatic identification and segmentation of bony structures in a CT scan of TPF. In addition to performing three-dimensional recognition and segmentation of healthy bones (femur, patella, tibia, and fibula), the algorithm aimed to detect TPF and differentiate individual fracture fragments.

## 2. Materials and Methods

### 2.1. Study Design

**Figure 1:**
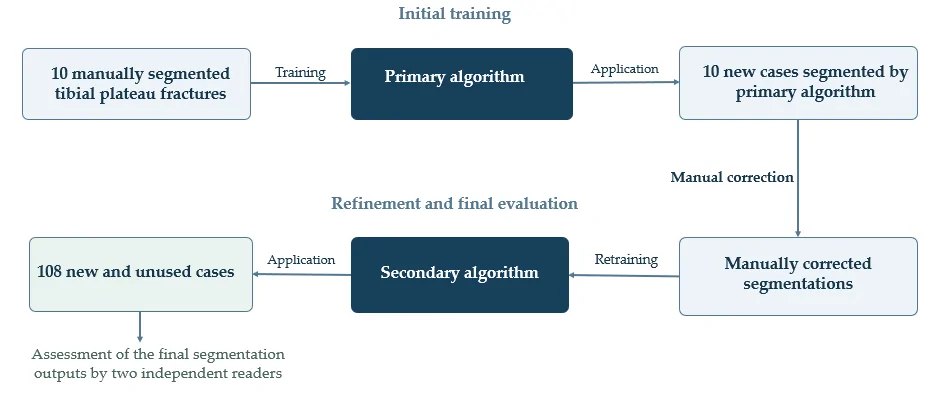
Flowchart demonstrating the creation pathway of the algorithm.

This single-center study involved analyzing CT scans of TPF using a fully automated, PACS-integrated software tool—based on a retrospective dataset of patients who underwent CT for surgery planning. For fully automatic analysis of the bones and fracture components, U-Nets were trained within a PACS (Visage Imaging GmbH, Berlin, Germany) using a set of 20 patients with TPF. The algorithm was trained in two phases. In the first phase, 10 CT scans of patients with TPF optimally segmented by two experienced readers (one radiologist with 9 years of experience and one orthopedic surgeon with 4 years of experience) were used to train an initial algorithm. In a second phase, the algorithm was applied to 10 additional fractures (Figure 1). These algorithm-generated segmentations were then manually refined by the readers and the algorithm was re-trained. The study received approval from the institutional review board.

### 2.2. Image Acquisition

Patients were either examined using a 128-multislice (Revolution HD and Revolution EVO, GE Healthcare, Milwaukee, WI, USA) or one of two 64-multislice CT scanners (Somatom Sensation 64, Siemens Medical Systems, Forchheim, Germany) with slice thickness ranging from 0.625 to 1.000 mm. All datasets were reconstructed multiparametrically using isotropic voxel generation. The relevant structures (femur, fibula, tibia, patella and fracture fragments) were manually segmented in consensus by two clinically experienced examiners: a radiologist with nine years and an orthopedic surgeon with four years of professional experience using a 3D contouring tool in Visage 7 (Visage Imaging GmbH, Berlin, Germany). Bones and individual fracture fragments were manually annotated slice by slice on CT images in the axial view. A bone component was considered a separate fracture fragment if it was visibly separated from the remaining bone. Coronal and sagittal views were used to support decisions regarding fragment boundaries.

For training, the data were exported in NIfTI format and the nnU-Net framework was used to train a 3D segmentation model [16]. For training and preprocessing, the default nnU-Net parameters were used. The framework automatically selected a patch size of 128 × 128 × 128 voxels with a voxel spacing of 0.8 × 0.8 × 0.625 mm along the left–posterior–superior (LPS) axes, respectively. For evaluation, the model was integrated into the Visage 7 using an interactive button which automatically segments the bones and fractures in the loaded CT dataset. No standardized measurement of processing time was performed. However, automated segmentation consistently took less than 60 s on a standard workstation routinely used in the orthopedic department in the daily clinical setting.

### 2.3. Description of the nnU-Net Framework

#### 2.3.1. Dataset Preparation, Fingerprint Extraction, and Configuration

First, the training images were cropped to their non-empty regions to reduce computational requirements. A “dataset fingerprint” was then extracted from the preprocessed data, describing relevant properties, including image dimensions, voxel spacing, intensity distributions, number of classes, imaging modality (CT), and number of training cases. In addition, the mean, standard deviation, and 0.5th and 99.5th percentiles of intensities within foreground regions were calculated. Based on the dataset fingerprint, nnU-Net automatically generated a “pipeline fingerprint” containing all parameters relevant to pipeline configuration. The framework distinguishes three categories of parameters:

1. Fixed parameters, such as the basic network architecture, learning rate, optimization algorithm, loss function, and data augmentation strategy, are adopted without modification.
2. Rule-based parameters, such as patch size, batch size, target voxel spacing, and resampling strategy, are derived from dataset properties using predefined heuristics.
3. Empirical parameters, such as ensemble selection and postprocessing, are selected through data-driven evaluation during model development.

#### 2.3.2. Network Architecture and Training Strategy

nnU-Net uses a U-Net-like encoder–decoder architecture with skip connections and instance normalization. Standard ReLU activations are replaced by leaky ReLU activations with a negative slope of 0.01. The networks incorporate deep supervision, with additional loss terms applied at intermediate decoder stages, to improve gradient flow. The number of feature maps starts at 32 and doubles at each downsampling stage, up to a maximum of 320 for 3D networks. Downsampling is performed using strided convolutions, whereas upsampling uses transposed convolutions.

#### 2.3.3. Resampling, Normalization, and Adaptation of the Network Architecture

To standardize voxel spacing, all images were resampled to a specified target spacing. For isotropic data, the framework uses third-order spline interpolation. For anisotropic data, spline interpolation is used in-plane and nearest-neighbor interpolation is used out-of-plane. CT intensities were clipped using the global 0.5th and 99.5th percentiles of foreground intensities and normalized using the global mean and standard deviation. The framework initially sets the patch size to the median image dimensions after resampling and progressively reduces it until the model can be trained with a batch size of at least two within the available GPU memory. During this process, the network architecture, including the number of pooling stages and convolutional kernel sizes, is automatically adjusted. If a patch in a 3D network covers less than 12.5% of the average image volume, a cascaded 3D U-Net configuration is generated. In this configuration, the first network operates on downsampled images, and its predictions are provided as additional inputs to a second network operating at high resolution.

#### 2.3.4. Inference, Postprocessing, and Implementation Details

During inference, a sliding-window approach with 50% overlap and Gaussian weighting toward the window centers was used to reduce transition artifacts. Test-time augmentation was performed by mirroring along all axes to improve robustness. The framework evaluates whether retaining only the largest connected components improves the mean Dice score on the cross-validation dataset and applies this postprocessing strategy only if an improvement is observed. nnU-Net was implemented in Python 3.8 using PyTorch 1.6.0. The batchgenerators library was used for data augmentation. The framework is available as an open-source tool [17].

### 2.4. Patient Population and Analyses

The study included randomly selected patients diagnosed with TPF at the Emergency Department of Charité University Hospital who were referred to the Department of Radiology between January 2012 and January 2023. A total of 108 patients with TPF were included in this study. The included cases were entirely separate from the 20 cases used for model training. Two independent readers (one orthopedic surgeon with 7 years of experience (R1), one radiologist with 9 years of experience (R2)) evaluated each case in three stages:

1. Assessment of intact bones to establish baseline anatomy.
2. Assessment of fracture detection and delineation by the algorithm.
3. Evaluation of fracture elements, specifically assessing the visibility and delineation of individual fracture components.

The purpose of the study was to evaluate the performance of an automated algorithm designed to detect and characterize tibial fractures. The readers’ assessments were used to evaluate algorithm performance.

Each evaluation was rated using a 5-point Likert scale, where higher scores indicated better visibility and diagnostic confidence. Each of the three criteria was evaluated using a 5-point Likert scale, with higher scores indicating better algorithm performance for the respective task: 1 = very poor; 2 = poor; 3 = moderate; 4 = good; and 5 = excellent. Ratings were based on the visibility and delineation of the respective anatomical structures or fracture components. For example, with regard to fracture-fragment differentiation, the rating scale was defined as follows:

1. Very poor visibility: fracture elements could not be reliably identified.
2. Poor visibility: only limited or uncertain recognition of fracture components.
3. Moderate visibility: fracture elements partially visible, but some uncertainty remained.
4. Good visibility: most fracture components clearly identifiable.
5. Excellent visibility: all fracture elements optimally visible and well delineated.

Inter-reader association and the algorithm’s performance were subsequently analyzed based on these ratings. The two readers were blinded to each other’s ratings. Fractures were classified according to the AO/OTA, Schatzker, and Moore classification systems by R1. Classifications were carried out in accordance with the original publications [18,19,20].

### 2.5. Statistical Analysis

Correlation analysis was undertaken by calculating Spearman’s rank correlation coefficients as well as Kendall’s Tau-b. Mean values as well as standard deviations were calculated. The normal distribution was tested via the Shapiro–Wilk test. Differences in ratings with regard to fracture types were analyzed using the Kruskal–Wallis test. A *p*-value < 0.05 was considered statistically significant. Data analysis was conducted using IBM SPSS Statistics Version 27 (IBM, Armonk, NY, USA) and Python Version 3.9.2 (Python Software Foundation, Wilmington, DE, USA).

## 3. Results

To assess the performance of the segmentation algorithm, three criteria were considered: (1) accuracy of segmenting intact bone structures, (2) detection of fractures, and (3) differentiation of individual fracture fragments. Each criterion was rated on a five-point Likert scale (higher scores indicating better visibility and higher diagnostic reliability) by two independent readers (R1 and R2). In addition to descriptive statistics, correlation analyses were conducted to quantify both inter-rater association and relationships between the rating criteria. Patient characteristics are featured in Table 1 while the distribution of fracture types are found in Table 2.

### 3.1. Descriptive Statistics (n = 108, No Missing Values)

**Figure 2:**
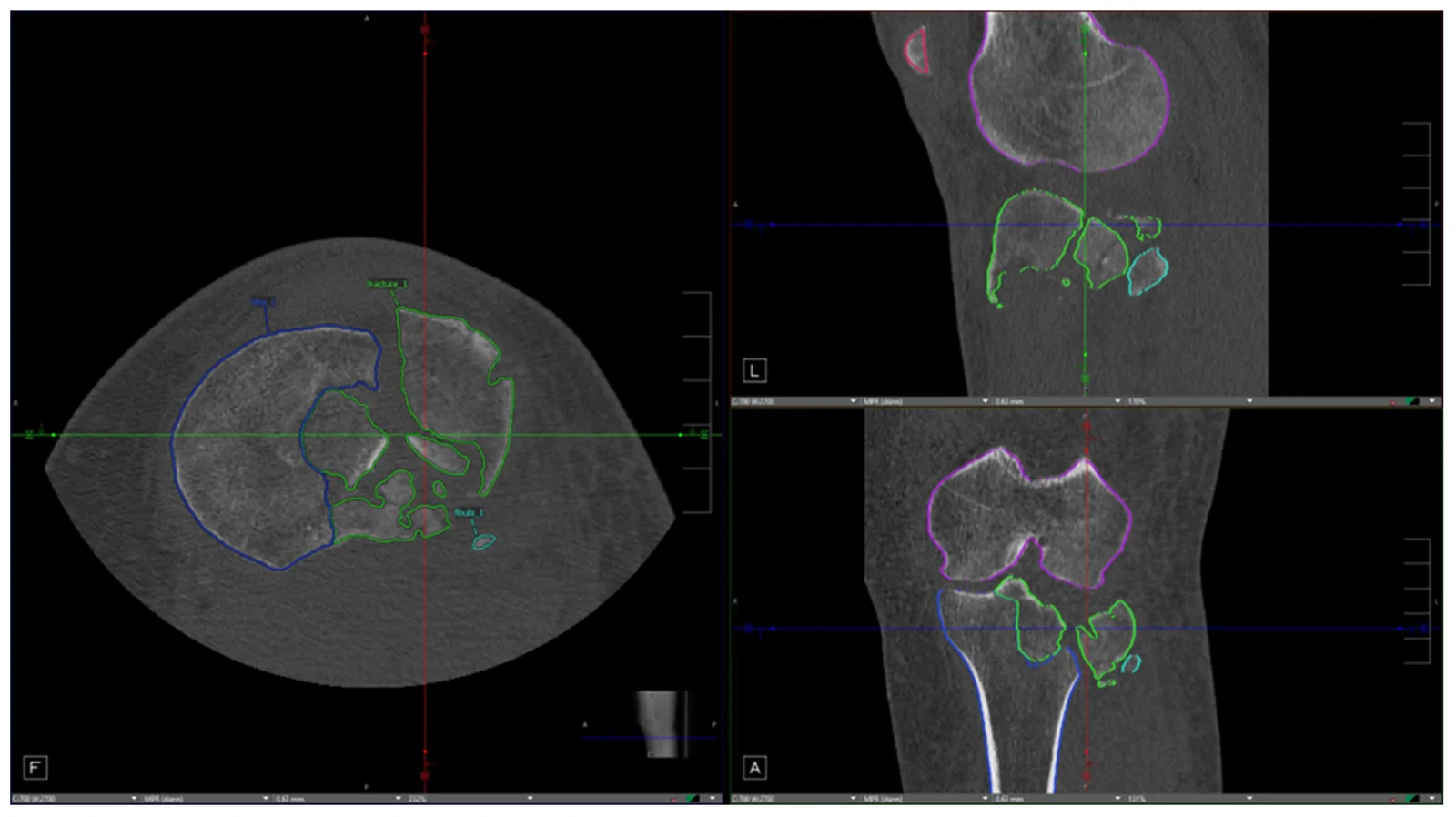
An example of a score of “2” for the distinguishability of individual fracture fragments—“limited visibility: only partial or uncertain recognition of individual fragments.” In the axial plane (left side of the image), it can be seen that the fracture fragments were correctly detected and even segmented separately from one another, but they are identified and labeled as a single fragment, even though there is no connection between the fragments.

**Figure 3:**
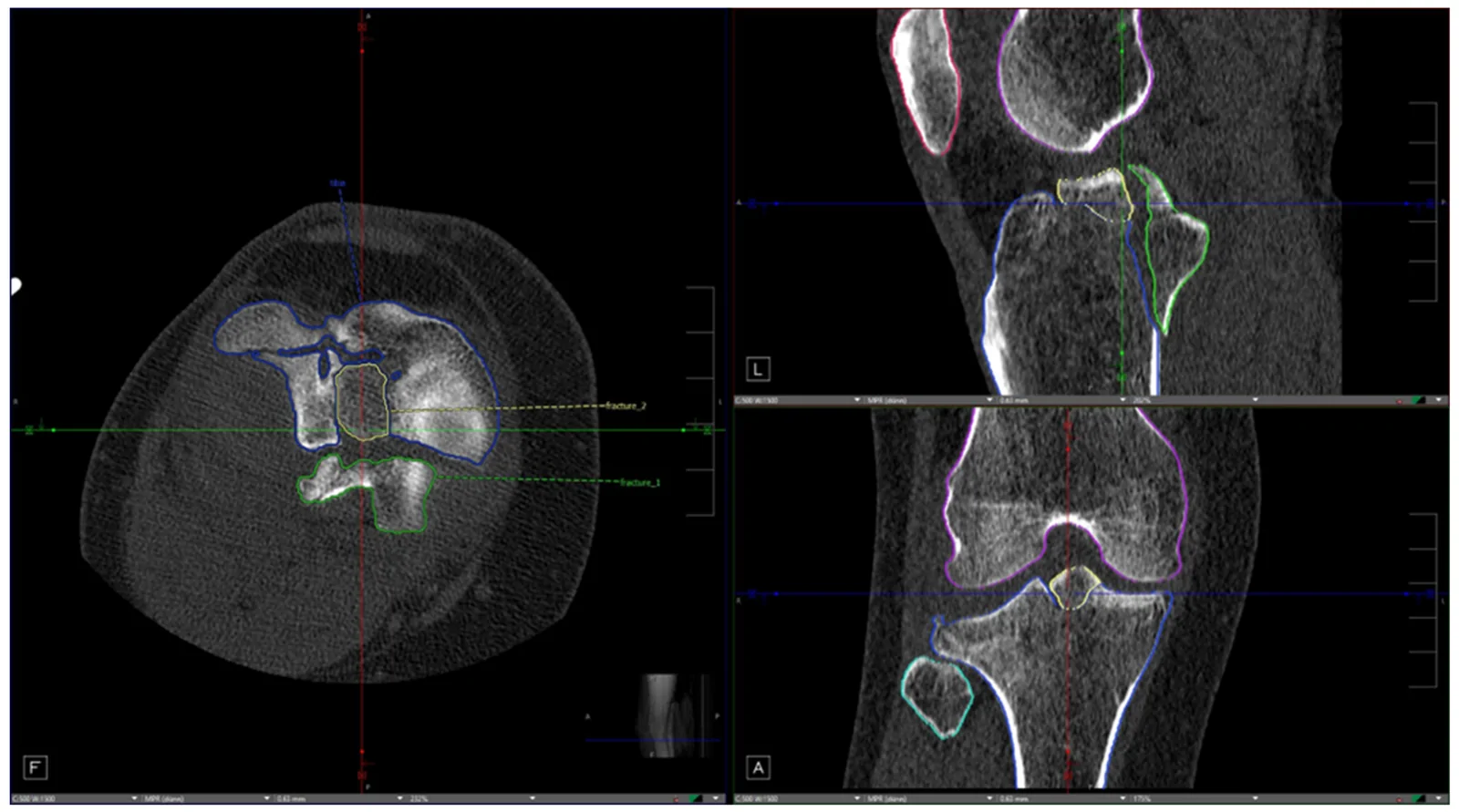
An example of a score of “5” for the distinguishability of individual fracture fragments—“excellent visibility: all fracture elements are optimally visualized and can be clearly delineated.” In the axial (left side of the image) and sagittal (top right) planes, the algorithm correctly segmented and displayed two well-separated fracture fragments (outlined in yellow and green).

For segmentation of healthy bone, ratings were very high, with mean scores of 4.67 (R1) and 4.72 (R2) and a median of 5.00 for both readers. Variability was low to moderate (SD 0.72 and 0.62, respectively), and the distributions showed a strong clustering at the maximum score (25th/50th/75th percentiles all 5.00). Fracture detection was also rated positively but slightly lower: mean scores of 4.15 (R1) and 4.20 (R2), median 4.00, with moderate variability (SD 0.88 and 0.81). In contrast, differentiation of individual fracture fragments was rated substantially lower: mean scores of 2.64 (R1) and 2.75 (R2) with a median of 2.00, variability was relatively broad (SD 0.94 and 0.93), and percentiles were 2.00 (25th/50th) and 3.00 (75th), indicating greater uncertainty and more heterogeneous case-by-case assessments. Representative examples are shown in Figure 2 and Figure 3. For all variables, the Shapiro–Wilk test indicated significant deviations from normality (*p* < 0.001), reflecting skewed Likert distributions (particularly a ceiling effect in the bone ratings).

### 3.2. Correlation Analyses (Inter-Rater Correlation)

Associations between R1 and R2 ratings were very strong across all criteria. For intact bone, a very strong positive rank correlation was observed (Spearman r = 0.952, df = 106, *p* < 0.001), along with very high rank-order association (Kendall’s tau-b = 0.958). For fracture detection, the strongest association in the entire analysis was found (Spearman r = 0.967, *p* < 0.001; tau-b = 0.971), indicating highly consistent identification of fracture lines by both experts. For fragments, correlation remained very high (Spearman r = 0.943, *p* < 0.001), with strong ordinal consistency (tau-b = 0.875), although, as expected, slightly lower due to the greater methodological complexity of fragment separation.

### 3.3. Analysis of Algorithm Performance According to Fracture Types

For the analysis of the performance of the algorithm with different fracture types we calculated the mean of the two reader ratings for each case. No significant differences in intact bone segmentation ratings were observed across fracture types.

Table 3 shows the average rating of the readers regarding the fracture detection performance of the algorithm in relation to fracture type.

**Table 3:** Differences in rating with regard to fracture detection and the different fracture types.

Fracture Classification	Fracture Type	n (%)	Mean ± SD
**AO/OTA classification**	A1	4 (3.7%)	4.50 ± 0.58
	B1	9 (8.3%)	4.11 ± 0.78
	B2	8 (7.4%)	4.25 ± 0.89
	B3	53 (49.1%)	4.32 ± 0.81
	C1	2 (1.9%)	4.00 ± 0.00
	C2	5 (4.6%)	4.00 ± 1.06
	C3	27 (25.0%)	3.89 ± 0.90
**Schatzker**	Type II	30 (35.7%)	4.50 ± 0.74
	Type III	7 (8.3%)	4.43 ± 0.79
	Type IV	11 (13.1%)	4.09 ± 1.04
	Type V	2 (2.4%)	4.00 ± 1.41
	Type VI	34 (40.5%)	3.88 ± 0.86
**Moore Classification**	Type I	6 (11.1%)	4.50 ± 0.84
	Type II	22 (40.7%)	4.18 ± 0.66
	Type IV	1 (1.9%)	4.00
	Type V	25 (46.3%)	4.00 ± 0.87

Although ratings varied across fracture types, with more complex fracture patterns such as AO/OTA type C3 and Schatzker type VI showing lower mean Likert scores for both readers, these differences did not reach statistical significance in the Kruskal–Wallis test (AO/OTA: *p* = 0.443; Schatzker: *p* = 0.063; Moore: *p* = 0.537). Ratings of fracture-fragment differentiation according to fracture type are shown in Table 4 and Table 5.

Lower ratings of algorithm performance were observed in AO/OTA C2- and C3-type fractures as well as Schatzker type VI fractures. These represent relatively complex fracture patterns with oftentimes multifragmentary fracture patterns. The Kruskal–Wallis test shown in Table 5 demonstrated that the differences within the AO/OTA and Schatzker classification systems were statistically significant with regard to fracture-fragment differentiation. The Moore classification did not show any significant differences in algorithm performance within the system.

## 4. Discussion

This study demonstrates the feasibility of an artificial intelligence (AI)-based, fully automated workflow integrated within a Picture Archiving and Communication System (PACS) for the identification and segmentation of bone structures and fracture fragments in computed tomography (CT) scans of tibial plateau fractures (TPF). The results underline the potential of AI to support trauma surgeons in developing optimal osteosynthesis plans by providing rapid, reproducible, and detailed anatomical and fracture-related information directly within the clinical imaging environment.

### 4.1. Performance of the Algorithm

The developed nnU-Net-based model achieved high ratings for identifying intact bone structures, with a median Likert score of 5.0, indicating near-perfect recognition of the femur, patella, tibia, and fibula. This performance aligns with the previous literature showing that CNN-based architectures perform robustly in anatomical segmentation tasks, particularly when trained on homogeneous datasets with high-quality annotations.

In contrast, the detection of fractures and differentiation of individual fragments presented more challenges. While fracture detection received high ratings (median 4.0), the algorithm’s ability to separate and label distinct fracture fragments was rated poorly (median 2.0). This finding suggests that the complexity and variability of tibial plateau fracture patterns, combined with the limited training dataset, still pose significant hurdles for automated segmentation. Nevertheless, the high Kendall rank correlation coefficients across readers (0.958, 0.971, and 0.875) indicate strong inter-observer ordinal association.

### 4.2. Interpretation of the Influence of Fracture Complexity on Algorithmic Performance

Our data show that more complex fracture patterns (e.g., AO/OTA B3, C2, and C3 type fractures as well as Schatzker type VI fractures) tend to lead to lower performance ratings of the algorithm, highlighting the limitations that exist in the delineation of fracture fragments. No statistically significant differences were observed within the Moore classification. Since the Moore classification was designed to describe fracture dislocations which generally represent more complex injury patterns, differences within this subgroup may be less pronounced. In general this may explain why no statistical significance was reached in this case. Furthermore the statistical power of this subgroup was limited due to the small subgroup sizes. No significant differences in fracture-detection ratings were observed across fracture types.

### 4.3. Clinical Implications

Integrating such an AI-based segmentation tool into PACS represents a major step toward real-time, clinician-friendly applications. Beetz et al. demonstrated the successful integration of an algorithm capable of fully segmenting images for CT-based body composition analysis, concluding that a rapid, fully automated, AI-driven assessment integrated into PACS can produce equivalent results without the need to transfer sensitive patient data [20]. To our knowledge, we are not aware of any current PACS-integrated CT algorithms available for automated fracture detection. There have been, however promising attempts at segmenting fracture fragments by deep learning [21], although previous studies report difficulties in accurately marking smaller fracture fragments in highly comminuted TPF situations [22,23]. By automatically generating 3D bone and fracture models, surgeons could potentially save time in preoperative planning and achieve better spatial understanding of fracture morphology [11].

### 4.4. Limitations

Despite the encouraging results, several limitations must be acknowledged. Most importantly this study relies on reader ratings but does not provide quantitative metrics such as Dice similarity coefficients or volumetric overlap ratios which could give more objective insights into segmentation performance. The training cohort was relatively small, comprising only 20 annotated cases, which may restrict generalizability to more complex or uncommon fracture patterns. This study only used a nnU-Net-based model in the creation of the algorithm and no alternative segmentation architectures were evaluated. Therefore no conclusion can be drawn with regard to the superiority of nnU-Net models over other segmentation approaches.

The fracture-type distribution was imbalanced, with almost half of the cohort classified as AO/OTA B3. The reliance on manual annotations from a limited number of readers introduces potential bias, and the test cohort, while larger, may not fully capture the spectrum of TPF morphologies encountered in clinical practice. Furthermore multiple fracture subgroups only contain a small number of cases. Therefore, the corresponding mean values should be rather interpreted as descriptive and not used to draw definitive conclusions with regard to algorithm performance between individual fracture groups. Manual segmentation was time-consuming; with a slice thickness of 0.625 mm, a 5 cm axial extent comprises approximately 80 CT slices requiring review and annotation. At the same time, complex fractures may comprise dozens of individual fracture fragments. This makes manual annotation of the fracture fragments highly time-consuming and also explains the relatively small number of primary cases available for training the algorithm which might have contributed to the results, especially in regard to poor fragment delineation in complex fractures. Nevertheless, high-quality datasets as ground truth are necessary to train robust algorithms.

### 4.5. Future Directions

Future work should focus on expanding the dataset to include a greater variety of fracture types, imaging conditions, and scanner models. Iterative refinement through additional training cycles, transfer learning, and the inclusion of synthetic or augmented data could improve fragment differentiation. The integration of biomechanical and spatial constraints might also enhance fracture segmentation fidelity. Ultimately, coupling the segmentation module with AI tools for fragment reduction and implant suggestion could transform the system into a comprehensive surgical planning assistant. Complex fractures in particular require careful preoperative planning. However, our findings suggest that the algorithm performs less well in delineating precisely these more complex fracture patterns while performing well in the detection of fractures as well as intact bone segmentation. This highlights the need for further refinement and validation of such algorithms before their implementation in routine clinical practice. As the system evolves, it may provide automated measurements or classifications, fragment reduction suggestions, and implant placement recommendations, further enhancing surgical decision-making. In principle, these algorithms could then also be applied to different locations across the human skeleton.

### 4.6. Conclusions

In summary, this study provides early evidence that a PACS-integrated AI-based workflow can automatically and reliably identify and segment intact bony structures and recognize fractures in TPF CT scans. However, exact fracture-fragment differentiation remained a challenge for the algorithm, especially in comminuted fractures. Continued algorithmic development and dataset expansion should aim to provide significant improvements, paving the way for clinical implementation and advancing the role of AI in orthopedic trauma imaging.

## Figures and Tables

**Table 1:** Patient characteristics of the analyzed cohort: continuous variables are presented as median [interquartile range]; categorical variables are presented as *n* (%). Data were available for age in 107 patients and for body weight, height, and BMI in 88 patients.

Characteristic	Value
Patients, *n*	108
Age, years	51 [40.5–57.5] (*n* = 107)
Male sex, *n* (%)	55/108 (50.9)
Female sex, *n* (%)	53/108 (49.1)
Body weight, kg	74.5 [66.0–89.5] (*n* = 88)
Height, cm	172.5 [164.3–180.0] (*n* = 88)
BMI, kg/m^2^	25.6 [22.5–28.9] (*n* = 88)

**Table 2:** Fracture distribution among the cohort. Percentages for the AO classification are based on all 108 fractures. Percentages for the Schatzker and Moore classifications are based on fractures applicable to the respective classification system (Schatzker: *n* = 84; Moore: *n* = 54).

Fracture Classification	Fracture Type	n (%)
**AO/OTA classification**	A1	4 (3.7%)
	B1	9 (8.3%)
	B2	8 (7.4%)
	B3	53 (49.1%)
	C1	2 (1.9%)
	C2	5 (4.6%)
	C3	27 (25.0%)
**Schatzker**	Type II	30 (35.7%)
	Type III	7 (8.3%)
	Type IV	11 (13.1%)
	Type V	2 (2.4%)
	Type VI	34 (40.5%)
**Moore Classification**	Type I	6 (11.1%)
	Type II	22 (40.7%)
	Type IV	1 (1.9%)
	Type V	25 (46.3%)

**Table 4:** Differences in rating regarding fracture-fragment delineation and the different fracture types.

Fracture Classification	Fracture Type	n (%)	Mean ± SD
**AO/OTA classification**	A1	4 (3.7%)	4.50 ± 1.00
	B1	9 (8.3%)	2.78 ± 1.09
	B2	8 (7.4%)	3.81 ± 1.19
	B3	53 (49.1%)	2.58 ± 0.77
	C1	2 (1.9%)	3.00 ± 0.00
	C2	5 (4.6%)	2.30 ± 0.45
	C3	27 (25.0%)	2.35 ± 0.59
**Schatzker**	Type II	30 (35.7%)	2.48 ± 0.72
	Type III	7 (8.3%)	3.79 ± 1.29
	Type IV	11 (13.1%)	3.14 ± 0.90
	Type V	2 (2.4%)	3.00 ± 1.41
	Type VI	34 (40.5%)	2.38 ± 0.56
**Moore Classification**	Type I	6 (11.1%)	2.75 ± 0.99
	Type II	22 (40.7%)	2.66 ± 0.90
	Type IV	1 (1.9%)	5.00
	Type V	25 (46.3%)	2.38 ± 0.60

**Table 5:** Kruskal–Wallis tests comparing fracture-fragment differentiation ratings across fracture types.

Classification System	H-Value (df)	*p*-Value
AO/OTA	**H(6) = 22.49**	**<0.001**
Schatzker	**H(4) = 15.50**	**0.0038**
Moore	H(4) = 4.44	0.2181

## Data Availability

The data presented in this study are available on request from the corresponding author due to local data privacy laws.

## Abbreviations

The following abbreviations are used in this manuscript:

AI	Artificial intelligence
CT	Computed tomography
TPF	Tibial plateau fracture(s)
PACS	Picture Archiving and Communication System
3D	Three-dimensional
NIfTI	Neuroimaging Informatics Technology Initiative
CNN	Convolutional neural network
LPS	Left–posterior–superior
SD	Standard deviation
R1	Reader 1
R2	Reader 2
df	Degrees of freedom
REPAIR	Recommendation for Evidence-based Preoperative AI-controlled virtual Reduction and osteosynthesis of complex fractures

## References

[1] Oeckenpöhler S., Domnick C., Raschke M., Müller M., Wähnert D., Kösters C. (2022). A lateral fracture step-off of 2mm increases intra-articular pressure following tibial plateau fracture. Injury.

[2] Meulenkamp B., Martin R., Desy N.M., Duffy P., Korley R., Puloski S., Buckley R. (2017). Incidence, Risk Factors, and Location of Articular Malreductions of the Tibial Plateau. J. Orthop. Trauma..

[3] Parkkinen M., Lindahl J., Mäkinen T.J., Koskinen S.K., Mustonen A., Madanat R. (2018). Predictors of osteoarthritis following operative treatment of medial tibial plateau fractures. Injury.

[4] Giannoudis P.V., Tzioupis C., Papathanassopoulos A., Obakponovwe O., Roberts C. (2010). Articular step-off and risk of post-traumatic osteoarthritis. Evidence today. Injury.

[5] Hak D.J., Rose J., Stahel P.F. (2010). Preoperative planning in orthopedic trauma: Benefits and contemporary uses. Orthopedics.

[6] Halai M., Hester T., Buckley R.E. (2020). Does 3D CT reconstruction help the surgeon to preoperatively assess calcaneal fractures?. Foot.

[7] Yao P., Gong M., Shan L., Wang D., He Y., Wang H., Zhou J. (2022). Tibial plateau fractures: Three dimensional fracture mapping and morphologic measurements. Int. Orthop..

[8] Ozturk A.M., Suer O., Derin O., Ozer M.A., Govsa F., Aktuglu K. (2020). Surgical advantages of using 3D patient-specific models in high-energy tibial plateau fractures. Eur. J. Trauma. Emerg. Surg..

[9] Krause M., Preiss A., Müller G., Madert J., Fehske K., Neumann M.V., Domnick C., Raschke M., Südkamp N., Frosch K.-H. (2016). Intra-articular tibial plateau fracture characteristics according to the “Ten segment classification”. Injury.

[10] Luo C.F., Sun H., Zhang B., Zeng B.-F. (2010). Three-column fixation for complex tibial plateau fractures. J. Orthop. Trauma..

[11] Assink N., Reininga I.H.F., Duis K.T., Doornberg J.N., Hoekstra H., Kraeima J., Witjes M.J.H., de Vries J.-P.P.M., Ijpma F.F.A. (2022). Does 3D-assisted surgery of tibial plateau fractures improve surgical and patient outcome? A systematic review of 1074 patients. Eur. J. Trauma. Emerg. Surg..

[12] Lo Mastro A., Grassi E., Berritto D., Russo A., Reginelli A., Guerra E., Grassi F., Boccia F. (2025). Artificial intelligence in fracture detection on radiographs: A literature review. Jpn. J. Radiol..

[13] Liu Y., Liu W., Chen H., Xie S., Wang C., Liang T., Yu Y., Liu X. (2023). Artificial intelligence versus radiologist in the accuracy of fracture detection based on computed tomography images: A multi-dimensional, multi-region analysis. Quant. Imaging Med. Surg..

[14] Kuo R.Y.L., Harrison C., Curran T.-A., Jones B., Freethy A., Cussons D., Stewart M., Collins G.S., Furniss D. (2022). Artificial Intelligence in Fracture Detection: A Systematic Review and Meta-Analysis. Radiology.

[15] Kahmann S.L., Rausch V., Plümer J., Müller L.P., Pieper M., Wegmann K. (2022). The automized fracture edge detection and generation of three-dimensional fracture probability heat maps. Med. Eng. Phys..

[16] Isensee F., Jaeger P.F., Kohl S.A.A., Petersen J., Maier-Hein K.H. (2021). nnU-Net: A self-configuring method for deep learning-based biomedical image segmentation. Nat. Methods.

[17] Meinberg E.G., Agel J.M., Roberts C.M., Karam M.D., Kellam J., Karam M. (2018). Fracture and Dislocation Classification Compendium-2018. J. Orthop. Trauma..

[18] Moore T.M. (1981). Fracture—Dislocation of the knee. Clin. Orthop. Relat. Res..

[19] Schatzker J., McBroom R., Bruce D. (1979). The tibial plateau fracture. The Toronto experience 1968–1975. Clin. Orthop. Relat. Res..

[20] Beetz N.L., Maier C., Segger L., Shnayien S., Trippel T.D., Lindow N., Bousabarah K., Westerhoff M., Fehrenbach U., Geisel D. (2022). First PACS-integrated artificial intelligence-based software tool for rapid and fully automatic analysis of body composition from CT in clinical routine. JCSM Clin. Rep..

[21] Liu Y., Yibulayimu S., Zhu G., Shi C., Liang C., Zhao C., Wu X., Sang Y., Wang Y. (2025). Automatic pelvic fracture segmentation: A deep learning approach and benchmark dataset. Front. Med..

[22] Kim H., Jeon Y.D., Park K.B., Cha H., Kim M.-S., You J., Lee S.-W., Shin S.-H., Chung Y.-G., Bin Kang S. (2023). Automatic segmentation of inconstant fractured fragments for tibia/fibula from CT images using deep learning. Sci. Rep..

[23] Yue P., Cai D., Yang X., Guo C., Liu M., Xia J., Wang Y. (2026). Learning generalizable features for bone fracture segmentation using limited annotations. Biomed. Signal Process. Control.

